# Analysis of *ZNF208* Polymorphisms on the Clinicopathologic Characteristics of Asian Patients with Hepatocellular Carcinoma

**DOI:** 10.7150/jca.98520

**Published:** 2024-08-13

**Authors:** Yi-Chung Chien, Hsiang-Lin Lee, Whei-Ling Chiang, Li-Yuan Bai, Yu-Ju Hung, Shuo-Chueh Chen, Hsiang-Ling Wang, Shun-Fa Yang, Yung-Luen Yu

**Affiliations:** 1 Institute of Translational Medicine and New Drug Development, China Medical University, Taichung 40402, Taiwan.; 2 Graduate Institute of Biomedical Sciences, China Medical University, Taichung 40402, Taiwan.; 3 Center for Molecular Medicine, China Medical University Hospital, Taichung 40402, Taiwan.; 4 School of Medicine, Chung Shan Medical University, Taichung 40201, Taiwan.; 5 Department of Surgery, Chung Shan Medical University Hospital, Taichung 40201, Taiwan.; 6 School of Medical Laboratory and Biotechnology, Chung Shan Medical University, Taichung 40201, Taiwan.; 7 Division of Hematology and Oncology, China Medical University Hospital, Taichung, 40402, Taiwan.; 8 Division of Pulmonary and Critical Care Medicine, Department of Internal Medicine, China Medical University Hospital, Taichung 40402, Taiwan.; 9 Department of Beauty Science, National Taichung University of Science and Technology, Taichung 404336, Taiwan.; 10 Institute of Medicine, Chung Shan Medical University, Taichung 40201, Taiwan.; 11 Department of Medical Research, Chung Shan Medical University Hospital, Taichung 40201, Taiwan.; 12 Department of Medical Laboratory Science and Biotechnology, Asia University, Taichung 41354, Taiwan.

**Keywords:** Hepatocellular Carcinoma, *ZNF208*, distant metastasis, single-nucleotide polymorphisms

## Abstract

Hepatocellular carcinoma (HCC), a major form of liver cancer, is characterized by high lethality and a multifactorial etiology that includes hepatitis virus infections, lifestyle factors, and genetic predispositions. This study aimed to explore the impact of *ZNF208* gene polymorphisms on the clinicopathological features of Taiwanese HCC patients, focusing on three specific single nucleotide polymorphisms (SNPs): rs2188971, rs2188972, and rs8105767. Our cohort consisted of 438 HCC patients and 1193 control individuals. Clinical staging was determined using the tumor/node/metastasis (TNM) system, and various clinical indicators were collected. Our analysis revealed a statistically significant increase in *ZNF208* expression in HCC patients compared to controls, indicating a potential role in HCC progression. Although no substantial association was observed between *ZNF208* SNPs and increased HCC risk, specific clinical features such as distant metastasis and vascular invasion showed significant associations with these SNPs, suggesting their influence on disease aggressiveness. Demographic analyses highlighted the importance of factors like alcohol consumption and viral hepatitis markers in HCC. Our study underscores the complexity of genetic influences on HCC, with *ZNF208* polymorphisms potentially affecting tumor progression and patient outcomes.

## Introduction

Liver cancer, mainly hepatocellular carcinoma (HCC), is marked by its high lethality and complex etiology involving hepatitis virus infections, non-alcoholic and alcoholic fatty liver disease, and genetic factors 1. The treatment landscape for HCC is multifaceted, ranging from surgical options for early stages to systemic therapies for advanced disease states. Surgical resection, local ablative techniques, and liver transplantation offer curative potential for early tumor stages. For unresectable or metastatic HCC, systemic therapies including sorafenib have been mainstays, with recent advancements introducing new first- and second-line treatment options, notably anti-PD-L1 combination therapies 2, 3. The stratification of liver cancer treatment hinges on the tumor stage and liver function, emphasizing the importance of personalized treatment plans. Techniques such as alcohol injection, chemoembolization, targeted drug therapy, immunotherapy, and supportive care are among the arsenal deployed against this malignancy​​​​. HCC is classified based on its molecular characteristics into three distinct subtypes known as iCluster 1, iCluster 2, and iCluster 3. These subtypes are delineated through integrative genomic analyses that include DNA mutations, copy number variations, and gene expression profiles. iCluster 1 is associated with metabolic reprogramming and the activation of the Wnt/β-catenin signaling pathway, frequently exhibiting CTNNB1 gene mutations, leading to constitutive β-catenin activation. iCluster 2 stands out with a strong immune signature, characterized by immune cell infiltration within the tumor microenvironment. Frequent alterations in the PI3K/AKT/mTOR pathway, such as PIK3CA mutations, are observed. iCluster 3 represents a more aggressive form of HCC, often linked to poor prognosis, characterized by chromosomal instability and a high mutation burden, including TP53 mutations. These mutations lead to defective DNA damage responses and genomic instability. 4-7. Furthermore, genetic variations play a significant role in the onset and progression of liver cancer, with single nucleotide polymorphisms (SNPs) being a focal point of research for understanding susceptibility and therapeutic responses. Research highlights the association of specific SNPs with increased risk for HCC. For example, a meta-analysis found no significant association between SNP TNF-α -1031 and HCC risk, demonstrating the complexity and need for further study in genetic contributions to liver cancer. Meanwhile, polymorphisms in the PNPLA3 gene were significantly associated with increased susceptibility to HCC in the Chinese Han population, underscoring the genetic predisposition element in liver cancer's etiology​​ 8. Additionally, polymorphisms in the VDR gene have been linked to the severity of liver disease and HCC risk, further illustrating the intricate relationship between genetics and liver cancer​​ 9. The effect of SNPs in lncRNA as ceRNA on HCC risk and prognosis also presents an interesting avenue for exploring genetic factors contributing to liver cancer​​ 10.

*ZNF208*, known as Zinc Finger Protein 208, is a gene that encodes a protein involved in the intricate process of gene regulation through DNA binding. This protein is part of the zinc finger protein family, characterized by their ability to bind to DNA and regulate gene transcription. The function of *ZNF208* is primarily associated with the regulation of transcription, DNA-templated processes, and the regulation of transcription by RNA polymerase II. These functions highlight its role in the transcriptional control mechanisms that are essential for proper cellular function and development 11. A comprehensive understanding of the molecular pathways involved in cancer, including those influenced by genes like *ZNF208*, is essential for developing effective treatments. An integrative approach that combines somatic mutation analysis with pathway analysis has been used to identify pathways linked with survival outcomes across various cancer types​​ 12. The investigation into the association of the *ZNF208* gene with various types of cancers, particularly esophageal and laryngeal cancers in the Chinese Han population, has unveiled significant findings. Research conducted across multiple studies reveals that specific single nucleotide polymorphisms (SNPs) within the *ZNF208* gene are linked to an increased risk of these cancers, suggesting a genetic predisposition influenced by variations in this gene. In esophageal cancer within the Chinese Han population, a connection between genetic variants in *ZNF208* and susceptibility to the disease has been identified. Specifically, SNPs such as rs8103163 and rs7248488 were associated with an increased risk of developing esophageal cancer 13. Similar to the esophageal cancer study, SNPs rs8103163 and rs7248488 in the *ZNF208* gene were found to contribute to susceptibility to laryngeal cancer. The research highlighted that individuals with the A allele of rs8103163 and the A allele of rs7248488 had an increased risk of developing laryngeal cancer 14. However, the precise impact of *ZNF208* gene polymorphisms on the progression and initiation of hepatocellular carcinoma (HCC) in the Taiwanese population has not been thoroughly explored. This study focuses on three specific polymorphisms within the *ZNF208* gene: rs2188971 and rs2188972, both located in the 3'-untranslated region (3'-UTR), and rs8105767, found within an intronic region. Our goal is to determine their association with HCC among Taiwanese patients and to assess their potential influence on the prognosis of this cancer.

## Materials and Methods

### Study Participants and Specimen Collection

In this research, a cohort of 438 patients with hepatocellular carcinoma (HCC) was assembled from the Chung Shan Medical University Hospital in Taichung, Taiwan, with all participants giving informed written consent at the time of enrollment. The clinical staging for these patients was determined at diagnosis using the tumor/node/metastasis (TNM) staging system advocated by the American Joint Committee on Cancer (AJCC, 2002). Diagnosis of liver cirrhosis was affirmed either through liver biopsy or abdominal ultrasound imaging. Data collection encompassed clinical indicators such as liver cirrhosis, levels of aspartate aminotransferase (AST), α-fetoprotein (AFP), alanine aminotransferase (ALT), tumor staging, dimensions, lymph node involvement, distant metastasis, hepatitis B virus surface antigen (HBsAg) status, and antibodies against hepatitis C virus (anti-HCV), which were extracted from medical records. A control group comprising 1,193 individuals, aged between 20 to 70 years and with no cancer history, was curated from the Taiwan Biobank. This group provided personal information regarding sex, age, smoking, and alcohol consumption habits, where consuming more than two alcoholic drinks daily qualified as significant alcohol use, and smoking a minimum of one cigarette daily over the past three months was categorized as regular smoking. The study protocol received approval from the Institutional Review Board of Chung Shan Medical University Hospital (CS2-21064).

### Comprehensive Analyses of *ZNF208* from The Cancer Genome Atlas (TCGA)

UALCAN stands as an intuitive and interactive web platform designed for the examination of cancer omics data, accessible at http://ualcan.path.uab.edu/index.html. It leverages TCGA level 3 RNA-seq alongside clinical data across 31 cancer types 15. Meanwhile, Gene Expression Profile Interactive Analysis 2 (GEPIA2), accessible at http://gepia2.cancer-pku.cn/#index, represents an enhancement of the original GEPIA. It facilitates the analysis of RNA-sequencing expression data from 9,736 tumor and 8,587 normal samples derived from both the TCGA and the GTEx project, employing a standardized processing pipeline 16. In our research, both UALCAN and GEPIA2 tools were utilized to conduct analyses concerning the differential expression between tumor and normal tissues, as well as to assess the impact of *ZNF208* expression on the overall survival of HCC patients.

### Selection of *ZNF208* Polymorphisms

In our investigation, we selected three specific single nucleotide polymorphisms (SNPs) within the *ZNF208* gene, based on data from the International HapMap Project. These include rs2188971 and rs2188972, both located within the 3'-untranslated region (3'-UTR), and rs8105767, found in an intronic region of the *ZNF208* gene.

### *ZNF208* Genotyping

The identification of *ZNF208* gene polymorphisms, specifically rs2188971, rs2188972, and rs8105767, was conducted using the ABI StepOne real-time polymerase chain reaction (PCR) system, equipped with SDS v3.0 software, both from Applied Biosystems, and utilized the TaqMan assay 17.

### Statistical Analyses

To assess the variations in age and demographic details between the HCC patient group and controls, we utilized the Mann-Whitney U test. Logistic regression models were employed to compute the odds ratios and their 95% confidence intervals (CIs), with a significance threshold set at a p-value less than 0.05. All statistical analyses were conducted utilizing SAS software.

## Results

### Expression Levels of *ZNF208* in Clinical Specimens and Its Impact on Survival Rates

**Figure 1:**
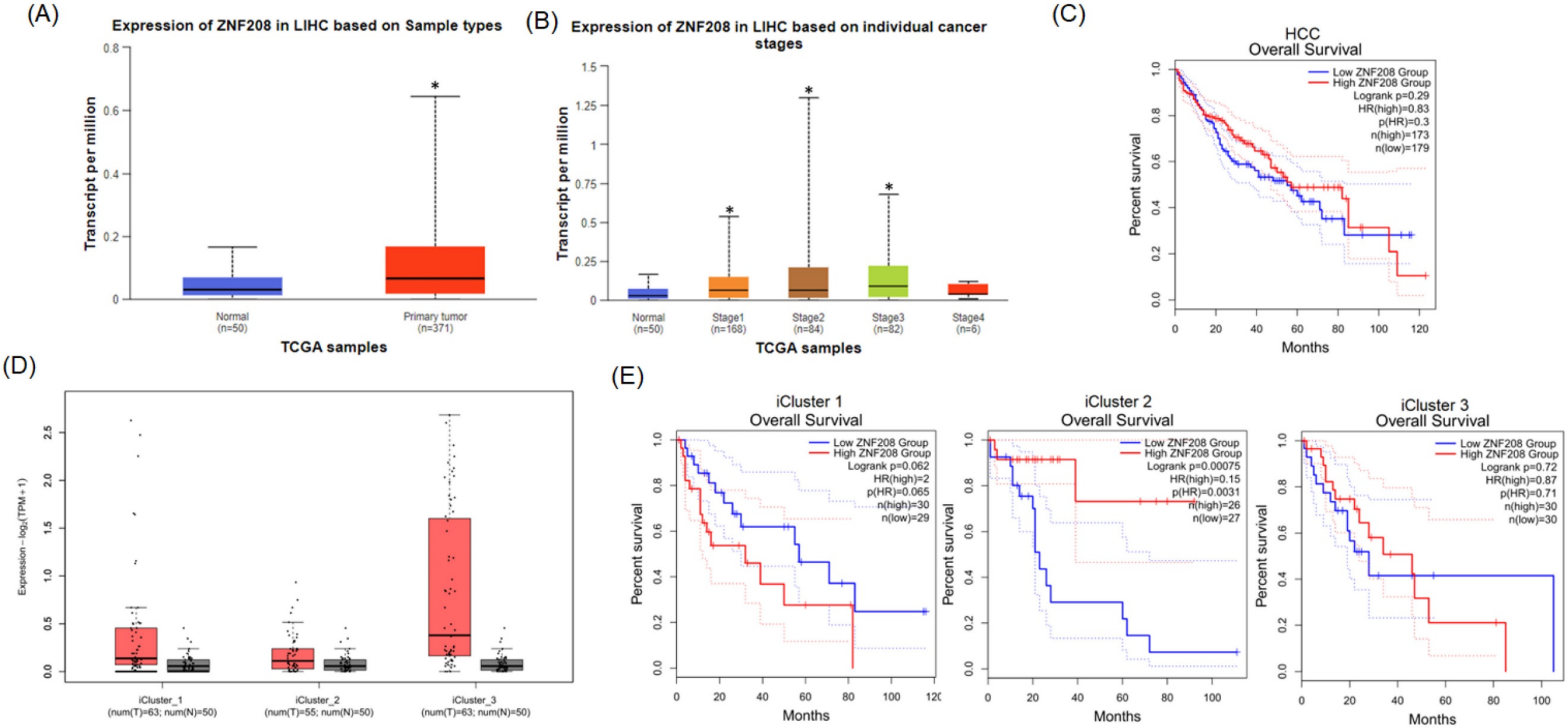
The level of *ZNF208* is correlated with HCC progression but not with the survival rate of HCC. (A) The level of* ZNF208* in normal control and hepatocellular carcinoma patients. (B) The level of *ZNF208* in normal control and different stages of HCC patients (C) The overall survival of different levels of *ZNF208* in HCC patients as assessed with data from GEPIA2. (D) The level of *ZNF208* in normal control and different HCC subtypes. (E) The overall survival of *ZNF208* in different HCC subtypes from GEPIA2. * p < 0.05

To ascertain the role and significance of *ZNF208* in HCC, we utilized UALCAN and GEPIA2 for analyzing HCC patient data from the TCGA database. Compared to healthy individuals, a statistically significant elevation in *ZNF208* expression was observed in HCC patient samples (Figure 1A). This upregulation suggests *ZNF208* may play a critical function in the progression of HCC. Further analysis of *ZNF208* expression across different HCC stages indicated a notable increase from stage one to three compared to normal samples (Figure 1B), implying a significant role for *ZNF208* in the early onset of HCC. Subsequent investigation into whether *ZNF208* expression levels affect the survival rates of HCC patients revealed no significant impact (Figure 1C). Furthermore, we examined the expression levels of *ZNF208* across different subtypes of HCC and their relationship with survival rates. The results indicated that there was no statistically significant difference in the expression levels of *ZNF208* between the different subtypes and the normal group (Figure 1D). However, upon subclassification into different subtypes, we observed that in the proliferative class, particularly within the iCluster 1 subtype, high *ZNF208* expression appeared to correlate with poorer survival rates; however, due to limited sample sizes, the statistical significance was marginal (p=0.065). Conversely, in the Non-proliferative class, iCluster 2 subtype, higher *ZNF208* expression was associated with improved patient survival and was statistically significant (Figure 1E). These findings indicate a complex regulatory mechanism of *ZNF208* in liver cancer, meriting further exploration to fully understand its implications in HCC pathogenesis and patient prognosis.

### Demographic Characteristics of Control Individuals and Hepatocellular Carcinoma Patients

To discern potential contributors to the onset of HCC in clinical settings, the demographic and clinical attributes of 1193 controls and 438 HCC patients were scrutinized in Table 1. No significant disparities were found in age groups, with those over 60 comprising 60.2% and 63.9% of the controls and HCC patients, respectively, nor in gender, with males representing approximately 70% in each cohort. Significant differences emerged in lifestyle factors; notably, alcohol consumption was higher among HCC patients, where 33.1% reported drinking compared to 14.1% of controls. Furthermore, HCC patients exhibited a higher incidence of HBsAg and anti-HCV markers, implying a strong linkage to the disease. The prevalence of liver cirrhosis was markedly higher in HCC patients (84%), and the majority were diagnosed at an early stage (I+II). Vascular invasion was noted in 13.5% of patients, indicating a degree of disease progression. These findings suggest alcohol consumption and viral hepatitis infections as significant factors associated with HCC in the clinical population analyzed.

### Associations of *ZNF208* genetic polymorphisms in HCC and normal controls

Next, we analyzed the genotypes and allele frequencies of *ZNF208* SNPs in a cohort of HCC patients and a control group. The genotypic distributions for the SNPs rs8105767, rs2188971, and rs2188972 revealed that the most common genotype in both controls and HCC patients was the homozygous reference genotype for each SNP. For rs8105767, the AA genotype was present in 45.4% of controls and 47.0% of patients. For rs2188971, the CC genotype was observed in 49.0% of controls and 49.5% of patients, while for rs2188972, the AA genotype was found in 26.7% of controls and 27.6% of patients. The odds ratios (ORs) and adjusted odds ratios (AORs), after accounting for age, gender, cigarette smoking, and alcohol drinking, did not show a significant association between the presence of these SNPs and an increased risk of HCC. The AORs were close to 1 for all genotypes across the three SNPs, indicating no substantial deviation from the reference in terms of increased or decreased risk for HCC. However, the results suggest that there might a slight genetic association between these specific *ZNF208* polymorphisms and the development of HCC, as reflected in this sample population.

### Association Between Clinical Status and *ZNF208* Genotypes rs8105767 and rs2188972 in HCC

To examine the correlation between clinical data and these *ZNF208* SNPs, our analysis focused on the genotypic frequencies of rs8105767 and rs2188972 in HCC patients. The findings revealed no significant correlation between the SNPs and the clinical stage when comparing early-stage (I/II) to advanced-stage (III/IV) HCC. However, significant associations emerged in specific clinical parameters. The *ZNF208* SNP rs8105767 was associated with a lower risk of HCV infection (p = 0.027) compared to wild-type patients. However, rs8105767 was linked to increased odds of distant metastasis and vascular invasion, indicating a potential association with more aggressive features of HCC. Specifically, distant metastasis was over twice as likely, and vascular invasion showed similarly increased odds in the presence of this SNP. Additionally, the rs2188972 SNP demonstrated a significant relationship with distant metastasis and vascular invasion, with nearly five times higher odds for distant metastasis and more than double the odds for vascular invasion, suggesting a potential link to advanced HCC progression. These significant associations between *ZNF208* SNPs and severe clinical manifestations of HCC suggest genetic variations may influence disease aggressiveness and patient outcomes, highlighting the need for further research to elucidate the role of these SNPs in HCC pathogenesis.

## Discussion

The amalgamation of broad genetic analyses and scholarly contributions underscores the pivotal role of single nucleotide polymorphisms (SNPs) in cancer susceptibility and prognosis. Research spanning various cancers, such as breast and gastric cancers, utilizes genome-wide and transcriptome-wide association studies to unearth new loci linked to cancer risk, unraveling the intricate ways SNPs affect gene regulation and cancer formation. A notable breast cancer study emphasized the necessity of considering the 3D genome structure for a deeper understanding of SNPs' regulatory impacts on gene expression within the cancer context 18. Concurrently, another investigation leveraged eQTL-weighted hierarchical Cox models for analyzing SNP-set based associations with cancer outcomes, shedding light on genetic variants' contribution to cancer risk and survival 19. Additionally, the systematic discovery of regulatory variants associated with cancer risk has showcased how SNPs within regulatory domains modulate gene expression, influencing cancer susceptibility and underscoring the functional significance of SNP in cancer biology​ 20. These discoveries are complemented by comprehensive reviews detailing the molecular and biological mechanisms by which SNPs impact gene expression, thus influencing cancer development and progression​​ 21. The link between SNPs and HCC has been explored through both case-control and prospective cohort studies, grounded in hypothesis-driven genetic research. These studies have highlighted how alterations affecting various biological pathways, such as inflammation, oxidative stress, iron metabolism, and DNA repair mechanisms, in patients with hepatitis are linked to the onset of liver cancer 22.

In our detailed analysis of *ZNF208* polymorphisms in HCC, we observed a notable increase in *ZNF208* expression among HCC patients compared to healthy controls, as shown in Figure 1, indicating the critical role of* ZNF208* in cancer progression. Our results also encompass demographic characteristics, genotyping, allele frequency of *ZNF208* SNP, and the association of these SNPs with clinical statuses in HCC patients. In Taiwan, a region where viral hepatitis prevalence is notably high, an elevated AST/ALT ratio often signifies underlying conditions such as chronic hepatitis B, hepatitis C, or fatty liver disease 23-26. Therefore, we also underscored factors like alcohol consumption and viral hepatitis markers significantly associated with HCC, highlighting the complex interplay of risk factors in cancer's etiology, as detailed in Table 1. Tables 2, 3, and 4 delve into the genotyping and allele frequencies of *ZNF208* SNPs, which, while not markedly deviating in risk for HCC, hint at minor genetic associations with the disease. Distant metastasis in HCC involves the spread of cancer cells from the liver to distant organs such as the lungs, bones, and lymph nodes. This metastatic process is complex and influenced by various genetic factors, including SNPs. For instance, polymorphisms in the melatonin receptor genes (MTNR1A and MTNR1B) have been associated with an increased risk of distant metastasis in HCC patients. Studies have shown that specific SNPs, such as rs2119882 and rs2375801 in MTNR1A, significantly correlate with an increased likelihood of metastasis, suggesting these polymorphisms may affect gene expression and function, thereby promoting cancer spread 27. Vascular invasion refers to cancer cells infiltrating blood vessels, facilitating the spread of the disease. SNPs in the VEGFA gene, which regulates vascular endothelial growth factor, play a significant role. Variants in VEGFA can lead to increased angiogenesis and vascular invasion, as polymorphisms such as -2578 C>A are significantly associated with the development and progression of HCC, highlighting their impact on tumor vascularization and invasiveness 28. Therefore, in our results, Table 3 elucidates the statistically significant associations of certain clinical features, like distant metastasis and vascular invasion, with these SNPs (rs2188971 and rs8105767). A study focusing on SNPs' association with gastric cancer susceptibility and prognosis further underscores the importance of genetic variations in cancer research, revealing that specific SNPs, such as rs4823921, significantly correlate with gastric cancer incidence and patient survival outcomes​ 29. The SNPs rs2188971 and rs8105767 within the *ZNF208* gene have been studied in the context of various cancers. For instance, SNPs like rs2188971 have been linked to increased cancer risk due to their potential impact on gene expression and function. These variations can alter the regulatory regions of *ZNF208*, thereby influencing its expression levels and downstream effects on cellular processes related to metastasis and invasion 13, 30. Our results found that increased levels of *ZNF208* in HCC are evident, the precise role of rs2188971 and rs8105767 SNPs in mediating distant metastasis and vascular invasion remains to be fully elucidated. However, some limitations exist. The sample size of this study might not be large enough to use propensity score matching (PSM), a statistical matching technique, to assess the intrinsic effects of *ZNF208* polymorphisms on the clinicopathologic characteristics. Future research should focus on disentangling these effects and should consider increasing sample size to provide clearer insights into their contributions to HCC pathogenesis.

Collectively, the employment of visual and statistical methodologies emphasizes the critical role genetic variations in cancer and marks our contributions to this burgeoning field. By exploring the association between *ZNF208* polymorphisms and HCC, our study adds to the growing evidence that SNPs significantly affect cancer susceptibility, tumor progression, and patient outcomes, necessitating additional research to unravel the mechanisms by which genetic variations influence HCC. This endeavor could have far-reaching implications for personalized cancer treatments and enhanced patient care.

## Conclusions

In summary, our discoveries indicate that genetic variations within *ZNF208* could serve as predictive markers for cancer susceptibility and hepatitis in HCC. This research contributes novel insights into the correlation between *ZNF208* polymorphisms and the clinical pathology of HCC within the Taiwanese population.

## Figures and Tables

**Table 1:** The distributions of demographical characteristics in 1193 controls and 438 patients with HCC.

Variable	Controls (N=1193)	Patients (N=438)	*p* value
Age (yrs)			
<60	475 (39.8%)	158 (36.1%)	*p*=0.169
≥ 60	718 (60.2%)	280 (63.9%)	
Gender			
Male	833 (69.8%)	309 (70.5%)	*p* = 0.777
Female	360 (30.2%)	129 (29.5%)	
Cigarette smoking			
No	724 (60.7%)	268 (61.2%)	*p* = 0.855
Yes	469 (39.3%)	170 (38.8%)	
Alcohol drinking			
No	1025 (85.9%)	293 (66.9%)	*p* < 0.001*
Yes	168 (14.1%)	145 (33.1%)	
HBsAg			
Negative	1048 (87.8%)	253 (57.8%)	*p* < 0.001*
Positive	145 (12.2%)	185 (42.2%)	
Anti-HCV			
Negative	1140 (95.6%)	256 (58.4%)	*p* < 0.001*
Positive	53 (4.4%)	182 (41.6%)	
Stage			
I+II		322 (73.5%)	
III+IV		116 (26.5%)	
Tumor T status			
T1+T2		329 (75.1%)	
T3+T4		109 (24.9%)	
Lymph node status			
N0		427 (97.5%)	
N1+N2+N3		11 (2.5%)	
Metastasis			
M0		412 (94.1%)	
M1		26 (5.9%)	
Vascular invasion			
No		379 (86.5%)	
Yes		59 (13.5%)	
Liver cirrhosis			
Negative		70 (16.0%)	
Positive		368 (84.0%)	

Mann-Whitney U test or Fisher's exact test was used between healthy controls and patients with HCC. * *p* value < 0.05 as statistically significant.

**Table 2:** Genotyping and allele frequency of *ZNF208* single nucleotide polymorphism (SNP) in HCC and normal controls.

Variable	Controls (N=1193) (%)	Patients (N=438) (%)	OR (95% CI)	AOR (95% CI)^a^
**rs8105767**				
AA	542 (45.4%)	206 (47.0%)	1.000 (reference)	1.000 (reference)
AG	525 (44.0%)	184 (42.0%)	0.922 (0.731-1.163)	0.888 (0.698-1.128)
GG	126 (10.6%)	48 (11.0%)	1.002 (0.693-1.450)	0.988 (0.676-1.443)
AG+GG	651 (54.6%)	232 (53.0%)	0.938 (0.753-1.168)	0.907 (0.723-1.137)
**rs2188971**				
CC	585 (49.0%)	217 (49.5%)	1.000 (reference)	1.000 (reference)
CT	502 (42.1%)	169 (38.6%)	0.908 (0.718-1.147)	0.917 (0.721-1.167)
TT	106 (8.9%)	52 (11.9%)	1.322 (0.917-1.908)	1.346 (0.922-1.965)
CT+TT	608 (51.0%)	221 (50.5%)	0.980 (0.787-1.220)	0.992 (0.792-1.243)
**rs2188972**				
AA	319 (26.7%)	121 (27.6%)	1.000 (reference)	1.000 (reference)
AG	600 (50.3%)	210 (47.9%)	0.923 (0.710-1.199)	0.884 (0.675-1.157)
GG	274 (23.0%)	107 (24.4%)	1.030 (0.758-1.398)	0.983 (0.717-1.348)
AG+GG	874 (73.3%)	317 (72.4%)	0.956 (0.748-1.222)	0.915 (0.710-1.179)

^a^ Adjusted for the effects of age, gender, cigarette smoking and alcohol drinking.

**Table 3:** Odds ratio (OR) and 95% confidence interval (CI) of clinical status and *ZNF208* rs8105767 genotypic frequencies in HCC patients.

Variable	Genotypic frequencies			
	AA (N=206)	AG+GG (N=232)	OR (95% CI)	*p* value
Clinical Stage				
Stage I/II	157 (76.2%)	165 (71.1%)	1.00	*p*=0.228
Stage III/IV	49 (23.8%)	67 (28.9%)	1.301 (0.848-1.997)	
Tumor size				
T1+T2	159 (77.2%)	170 (73.3%)	1.00	*p*=0.345
T3+T4	47 (22.8%)	62 (26.7%)	1.234 (0.797-1.909)	
Lymph node metastasis				
No	201 (97.6%)	226 (97.4%)	1.00	*p*=0.915
Yes	5 (2.4%)	6 (2.6%)	1.067 (0.321-3.550)	
Distant metastasis				
No	199 (96.7%)	213 (91.8%)	**1.00**	*p*=0.034*
Yes	7 (3.4%)	19 (8.2%)	**2.536 (1.044-6.162)**	
Vascular invasion				
No	189 (91.7%)	190 (81.9%)	**1.00**	*p*=0.003*
Yes	17 (8.3%)	42 (18.1%)	**2.458 (1.351-4.470)**	
HBsAg				
Negative	121 (58.7%)	132 (56.9%)	1.00	*p*=0.697
Positive	85 (41.3%)	100 (43.1%)	1.078 (0.737-1.577)	
Anti-HCV				
Negative	109 (52.9%)	147 (63.4%)	**1.00**	*p*=0.027*
Positive	97 (47.1%)	85 (36.6%)	**0.650 (0.443-0.952)**	
Liver cirrhosis				
Negative	36 (17.5%)	34 (14.7%)	1.00	*p*=0.421
Positive	170 (82.5%)	198 (85.3%)	1.233 (0.739-2.057)	

The ORs with analyzed by their 95% CIs were estimated by logistic regression models. * *p* value < 0.05 as statistically significant.

**Table 4:** Odds ratio (OR) and 95% confidence interval (CI) of clinical status and *ZNF208* rs2188972 genotypic frequencies in HCC patients.

Variable	Genotypic frequencies			
	AA (N=121)	AG+GG (N=317)	OR (95% CI)	*p* value
Clinical Stage				
Stage I/II	96 (79.3%)	226 (71.3%)	1.00	*p*=0.088
Stage III/IV	25 (20.7%)	91 (28.7%)	1.546 (0.935-2.557)	
Tumor size				
T1+T2	97 (80.2%)	232 (73.2%)	1.00	*p*=0.131
T3+T4	24 (19.8%)	85 (26.8%)	1.481 (0.888-2.469)	
Lymph node metastasis				
No	120 (99.2%)	307 (96.8%)	1.00	*p*=0.164
Yes	1 (0.8%)	10 (3.2%)	3.909 (0.495-30.866)	
Distant metastasis				
No	119 (98.3%)	293 (92.4%)	**1.00**	*p*=0.019*
Yes	2 (1.7%)	24 (7.6%)	**4.874 (1.134-20.947)**	
Vascular invasion				
No	112 (92.6%)	267 (84.2%)	**1.00**	*p*=0.022*
Yes	9 (7.4%)	50 (15.8%)	**2.330 (1.108-4.900)**	
HBsAg				
Negative	75 (62.0%)	178 (56.2%)	1.00	*p*=0.269
Positive	46 (38.0%)	139 (42.8%)	1.273 (0.829-1.955)	
Anti-HCV				
Negative	62 (51.2%)	194 (61.2%)	1.00	*p*=0.059
Positive	59 (48.8%)	123 (38.8%)	0.666 (0.437-1.016)	
Liver cirrhosis				
Negative	19 (15.7%)	51 (16.1%)	1.00	*p*=0.922
Positive	102 (84.3%)	266 (83.9%)	0.972 (0.547-1.725)	

The ORs with analyzed by their 95% CIs were estimated by logistic regression models. * *p* value < 0.05 as statistically significant.
